# Gender Dysphoria and Related Symptoms in Autism Spectrum Disorder: A Bilingual Review of the Literature

**DOI:** 10.3390/brainsci14121202

**Published:** 2024-11-28

**Authors:** Masaru Tateno, Ryotaro Shimomura, Eri Shiraishi, Kotaro Nanba, Yukie Tateno

**Affiliations:** Tokiwa Child Development Center, Tokiwa Hospital, Tokiwa 3-1-6-1, Minami-ku, Sapporo 0050853, Japan; rshimomura1119@gmail.com (R.S.); erieri-o@f3.dion.ne.jp (E.S.); yukimasata@yahoo.co.jp (Y.T.)

**Keywords:** autism spectrum disorder, neurodevelopmental disorders, gender dysphoria, gender incongruence, gender nonconformity, gender variance, gender diversity, transgender

## Abstract

The association between autism spectrum disorder (ASD) and gender dysphoria (GD) has attracted a great deal of interest among child and adolescent psychiatrists in Japan. In clinical settings, it is common to hear complaints or concerns related to GD from adolescents with ASD. In the past few years, several review articles have been published on ASD and GD. The Initial Clinical Guidelines for co-occurring ASD and GD in adolescents were published in 2018, suggesting the increasing need of intervention for these conditions worldwide. Although a large amount of evidence has been accumulated regarding the co-occurrence of ASD and GD, all review articles were based solely on case reports and articles published in English. In this article, we performed a bilingual literature review using English- and Japanese-language literature databases. We found 13 case reports in English and 11 case reports in Japanese. The Japanese literature included articles on gender-related symptoms in ASD, but not limited to ASD with comorbid GD. Wattel and her colleagues proposed 15 theories on the link between ASD and GD. We classified the reported cases into one or more of the fifteen theories proposed by Wattel. These theories seemed useful in understanding the co-occurrence of ASD and GD, especially in AMAB cases. Wattel’s 15 theories are categorized into biological, psychological, and social factors, respectively. With regard to the social factors, we discussed Japanese school culture and psychological burden among gender-dysphoric students. Further studies are awaited.

## 1. Introduction

The association between autism spectrum disorder (ASD) and gender dysphoria (GD) is one of the concerns among medical and welfare professionals who work with adolescents with mental health issues. In the fifth edition of the *Diagnostic and Statistical Manual of Mental Disorder*, ASD is defined as one of various neurodevelopmental disorders (NDDs), along with attention deficit/hyperactivity disorder (AD/HD) and intellectual development disorder (ID) [1]. Because NDDs often co-exist with one another, with wide range of severity, the clinical picture of each child can be extremely diverse. Children with ASD continue to develop along unique developmental trajectories rather than remain at any single developmental stage [2]. While we, as medical professionals, continuously support children with ASD, they sometimes exhibit psychological confusion in adolescence, a time during which the establishment of gender identity is one of the important psychosocial developmental tasks.

According to the diagnostic criteria of *DSM-5*, ASD is characterized by (1) persistent deficits in social communication and social interaction across multiple contexts and (2) restricted, repetitive patterns of behavior, interests, or activities. Recently, some experts have encouraged the use of the term neurodiversity, with respect to viewing ASD as an aspect of identity rather than a significant deficit [3,4]. It is known that people with ASD often have one or more comorbid psychiatric disorders, as the features of ASD cause various difficulties in daily life [5,6]. ASD often has comorbid internalizing disorders such as depression and anxiety, but an occasional comorbidity is GD [5,7,8].

Around year 2000, the prevalence of ASD was reported to be 1% [9]. Over the past two decades, however, the prevalence has increased steeply [10]. A recent review article by Gallin et al. reported the prevalence of ASD as about 2% worldwide [11]. The prevalence of ASD in Japan was reported as 1.8% in the 2000s, much higher than that reported from other countries at that time [12]. More recent results from large-scale surveys in Japan have reported a prevalence of 3.2% among 5-year-olds in Hirosaki city, Aomori, and 3.1% among 32-month-olds in Hamamatsu city, Shizuoka [2,13]. The latest report by the Autism and Developmental Disabilities Monitoring (ADDM) Network, in the U.S., also reported a prevalence of ASD of 2.8% [14]. Based on these reports, the incidence of ASD should be considered to be about 3%. An increase in the prevalence of ASD could also mean an increase in ASD with comorbid GD.

Japan has a well-organized health check-up system for 18- and 36-month-old infants [15]. The relatively higher prevalence of ASD in Japan may be a result of the health check-up system for infants. Recent data report that the examination rate exceeds 94% for both 18- and 36-month-old infants [16]. This means that early intervention is possible for infants with significant signs of ASD from early childhood. Depending on the severity of ASD features and the need for medical assistance, children with suspected developmental delays will be referred to child psychiatry clinics by the local health care center. In Japan, ASD cases generally continue to visit medical institutions from infancy to adolescence or adulthood, although there are differences among regions, and there is a serious shortfall of child psychiatry medical institutes [17]. Continuous support of autistic children from infancy through adolescence as a child and adolescent psychiatrist means that we are able to observe them facing identity fluidity and their struggle to establish identity in adolescence. During adolescence, autistic children may experience identity fluidity that could be related to ASD traits, and this nascent uncertainty of identity may develop into gender dysphoria.

Nowadays, the relationship between ASD and gender dysphoria has attracted a great deal of interest among child and adolescent psychiatrists in Japan. In clinical settings, it is common to hear complaints or concerns related to gender dysphoria from adolescents with ASD [18]. However, the co-occurrence of ASD and GD is often difficult to deal with in a general outpatient setting. The shortage of gender-specific clinics is also a serious problem. The interest in the co-occurrence of ASD and GD seems to be a worldwide trend. In 2018, initial clinical guidelines for co-occurring ASD and GD in adolescents were published, which were very helpful for clinicians, and their content was embraced worldwide [19]. In the past few years, several review articles have been published on ASD and GD [20,21,22,23,24,25,26,27,28,29,30,31,32,33]. Although a large amount of evidence has been accumulated regarding the co-occurrence of ASD and GD, all review articles were based solely on case reports and articles published in English. Although socio-cultural factors might be linking ASD and GD, most researchers on this topic have limited access to research databases in a language other than English. Thus, in this article, we performed a bilingual literature review using ICHUSHI (https://www.jamas.or.jp/english/, accessed on 30 September 2024), the largest Japanese-language literature database, to identify autistic cases with comorbid GD as well as ASD with gender-related symptoms, in addition to conducting a literature search using English-language literature databases.

Numerous studies have reported a strong association between ASD and GD, especially in adolescents. We believe that a consensus has already been reached on the relationship between these two conditions. Although a lot of papers have been published, the studies’ designs could be divided into three main categories: (1) the occurrence of GD and GD-like conditions in ASD, (2) the occurrence of ASD and ASD traits in people with GD and GD-like traits, and (3) an investigation of ASD traits and GD traits using self-administered questionnaires in the general population. In addition, some studies have asked caregivers about their children’s transgender-related behaviors by having them complete the Child Behavior Checklist (CBCL) [34,35,36,37,38]. The CBCL consists of 113 questions in 8 categories including 2 items related to possible transgender identification: “behaves like opposite sex” and “wishes to be opposite sex”. Despite the variety of ages of study subjects and research methods, many studies have demonstrated consistently a significant link between ASD and GD. However, the underlying mechanism that links these two conditions has not been well explored.

Gender-dysphoric adolescents often have psychiatric comorbidities such as depression and/or anxiety disorder [39,40,41]. Some cases of school refusal seem to be related to GD, especially in junior high school and high school. The first author and other members of the GID work group of the Japanese Society of Child and Adolescent Psychiatry (https://child-adolesc.jp/, accessed on 30 September 2024) investigated clinical practices of child and adolescent psychiatrists who may have treated adolescents with GD and gender identity-related concerns in Japan [18]. Those results demonstrated that, even among experienced child and adolescent psychiatrists, the breadth of understanding diagnosis and interventions for students with GD is limited. Among the 128 respondents, 74 (57.8%) had seen children and adolescents with *DSM-5* GD, and 87 (67.7%) had examined cases with gender identity-related concerns. However, the mean number of career-spanning experience with cases of GD was only 1.80 ± 2.3 per respondent.

In this paper, we focused on GD and gender-nonconforming symptoms in ASD and reviewed case studies reported in both English and Japanese by performing literature searches in both languages. As child and adolescent psychiatrists, the authors see many autistic adolescents. Some of them occasionally complain of gender-dysphoric feelings. However, because their gender-nonconforming symptoms are not always persistent, it is often difficult for us to provide them with appropriate support. In addition, due to the lack of specialized gender clinics in Japan, it is almost impossible for us to support gender-dysphoric autistic people in collaboration with a GD specialist. Many adolescents with ASD show school refusal after entering junior high school. The primary reason for this is likely to be the difficulty in adapting to peer groups due to social impairment as one of the clinical features of ASD. However, some junior high and high school students with ASD will have difficulty in attending school due to the psychological distress caused by wearing a school uniform based on their assigned gender at birth. It is possible that social factors in Japan that require junior high and high school students to wear gender-specific school uniforms may be related to gender-nonconforming symptoms in ASD. Therefore, in order to provide better intervention for them, the authors reviewed previous studies on ASD and GD in both English and Japanese to understand why gender-nonconforming symptoms are more common in autistic individuals than in typically developing individuals.

The purpose of this study is to investigate the empirical validity of the “theories” proposed to explain the association between ASD and GD. The universality of the “theories” will also be examined by comparing case reports published from various countries with different cultural backgrounds with those reported in Japan.

The terms used to describe the condition of feeling significant discomfort with one’s assigned gender vary according to the year of publication of the articles due to the revision of the *Diagnostic and Statistical Manual of Mental Disorders* (DSM) and the International Classification of Diseases (ICD). As the term “gender dysphoria” in the *DSM-5* [1] is considered to represent a concept that encompasses “gender identity disorder” (GID) in the *DSM-IV* [42], we employ the term GD consistently throughout this paper.

## 2. Materials and Methods

### 2.1. Literature Search

#### 2.1.1. Literature Search in English

We performed a literature search following previous review studies [20,23,25] conducted in accordance with the PRISMA guideline [43].

Initially, we searched databases for English literature, such as PubMed, from March 2024 to July 2024, repeating the search processes every month to ensure that there were no omissions in the targeted papers.

Terms for ASD (autism, autistic, autism spectrum disorder, pervasive developmental disorder, and Asperger) and GD (gender dysphoria, gender-dysphoric, gender dysphori*, gender incongruence, gender identity disorder, gender identity, transgender*, transgender, transsex*, and crossdressing) were used in the title and abstract search fields. Search terms were combined using “AND” and “OR” operators.

#### 2.1.2. Literature Search in Japanese

We also performed a literature search using a Japanese literature database named ICHUSHI (www.jamas.or.jp/english/, accessed on 29 July 2024) during the same period. ICHUSHI is the largest Japanese-language literature database, containing more than 16 million items of academic literature, including abstracts of oral and poster presentations at domestic congresses.

We used the Japanese terms for ASD (autism, autistic, autism spectrum disorder, pervasive developmental disorder, and Asperger*) and GD (gender dysphoria, gender-dysphoric, gender dysphori*, gender incongruence, gender identity disorder, gender identity, transgender*, transgender, transsex*, crossdressing, sexual minority, and LGBT) for the “Title and Abstract” search. Further searches were conducted by combining the search results using “AND” and “OR” Boolean operators.

### 2.2. Inclusion Criteria for This Review

In this study, we included papers that had sufficient descriptions of symptoms to determine which theory was better at explaining the link between ASD and GD-related symptoms. In general, those with symptom descriptions of 1000 Japanese characters or more were included. After a literature search by two of the authors (M.T., R.S.), five authors (M.T., R.S., E.S., K.N., and Y.T.) read all the papers listed as candidate case reports. Finally, all the authors discussed and decided whether or not each case report was appropriate for inclusion in this study.

### 2.3. Classification of Underlying Theories

In 2016, van der Miesen and her colleagues suggested eight underlying hypotheses distinguishing between ASD and GD [25]. Based on a detailed review of the numerous papers reporting the relationship between ASD and GD, Wattel et al. proposed fifteen theories that may link these two conditions with reference to van der Miesen’s hypotheses [26].

Initially, we reviewed published case reports of ASD with a confirmed diagnosis of GD or GD-related symptoms to investigate whether van der Miesen’s hypotheses could be applied as possible explanations for our cases. In the meantime, Wattel et al. [27] proposed fifteen theories based on the eight theories previously proposed by van der Miesen [25]. Some of our provisional explanations were similar to theories proposed by Wattel et al. [27]. Therefore, the authors decided to refer to Wattel’s 15 theories to understand the relationship between ASD and GD, rather than proposing a new explanation.

We classified the ASD cases with confirmed diagnosis of GD or with GD-related symptoms reported in Japan based on the theories demonstrated by Wattel et al. [27]. The classification of each case was based on the consensus of all the authors of this paper.

## 3. Results

### 3.1. Literature Search

#### 3.1.1. Literature Search in English

The final literature search of the English database was performed on 27 September 2024. A large number of papers were obtained for each search term. The following are the search results for terms related to ASD: autism (68,669 results), autism spectrum disorder (31,536 results), autistic (18,803 results), Asperger (2410 results), and pervasive developmental disorder (1311 results). A combined search of these terms using the Boolean operator “OR” generated 74,866 results. Similarly, the search results for terms related to GD were as follows: gender dysphoria (2220 results), gender-dysphoric (197 results), gender dysphori* (2310 results), gender incongruence (390 results), gender identity disorder (516 results), gender identity (6269 results), transgender* (14,522 results), transgender (14,258 results), transsex* (2788 results), and crossdressing (167 results). By combining these terms with “OR”, a total of 20,743 papers were found. We performed a combined search of ASD and GD-related terms using the Boolean operator “AND”, which selected 242 results. Filtering these results by “Case Reports” and “English” provided 13 articles [44,45,46,47,48,49,50,51,52,53,54,55,56]. These cases had already been listed and investigated thoroughly in a review paper by van der Miesen [26], except for the cases reported by Zupanič and Sheikhmoones [55,56]. The cases are listed in Table 1. Based on the symptomatic descriptions of each case report, we examined which of the theories proposed by Wattel [27] could explain them. The abbreviation AMAB was used to signify that the gender assigned at birth was male, and AFAB was used if the gender assigned at birth was female [57].

#### 3.1.2. Literature Search in Japanese

The following search terms were entered in the “Title/Abstract” field, like in the English literature search. The number of search results for each term is indicated in parentheses: autism (12,166 results), autism spectrum disorder (3624 results), autistic (17,600 results), Asperger* (2807 results), and pervasive developmental disorder (3368 results). A combined search of these terms using the Boolean operator “OR” generated 22,496 results. Similarly, the search results for terms related to GD were as follows: gender dysphoria (416 results), gender-dysphoric (106 results), gender incongruence (86 results), gender nonconformity (13 results), gender identity disorder (1829 results), gender identity (1921 results), crossdressing (8 results), sexual minority (8 results), LGBT (647 results), and transgender (313 results). In total, 2962 GD-related abstracts were found. The combined search for ASD and GD-related terms using the Boolean operator “AND” screened 66 abstracts. After excluding congress abstracts (oral or poster presentation at congresses), 43 papers remained for further survey. Of these 43 papers, only 11 case reports had case descriptions. Of them, one of the case reports by Ito et al. was excluded from further analysis because it was a secondary abstract of a presentation at a Japanese conference, and there was insufficient information about the case [58]. Meanwhile, the cases reported by the first author were reported in this paper with additional information by chart review [59,60,61,62,63,64,65,66,67,68]. The cases are summarized in Table 2.

### 3.2. Classification of Japanese Cases Based on the Underlying Theories Proposed by Wattel

All case reports included in this review were read by all the authors independently. To classify the cases into one or more of the fifteen theories regarding the link between ASD and GD proposed by Wattel [27] (Table 3), all the authors discussed the cases, and their classification was decided by consensus.

Brief descriptions of the Japanese cases and applicable theories proposed by Wattel are summarized in Table 4.

We attempted to classify the reported cases with ASD and GD based on Wattel’s theory from the information provided in the articles. Because of the limited description of symptoms in some articles, the application of the theory to each case was based on the interpretation that we found most plausible. Many of the gender-nonconforming symptoms in ASD reported in Japan could be explained by one or more of Wattel’s theories. Similar trends in the applied theories were found when comparing case reports from other countries with Japanese cases.

The extreme male brain theory was applicable only to AFAB and not to AMAB. Symptoms related to sensory processing are associated with unpleasant tactile sensations caused by body hair. Thus, sensory processing theory may be more applicable to AMAB than AFAB. Accordingly, these theories are unable to account for the correlation between ASD and GD in all instances.

While there is a possibility that sexual development may be associated with intelligence levels, the information available on the intelligence quotient (IQ) was often limited in the case reports. Obsession was referenced in many case reports in both English and Japanese. As Wattel’s review demonstrated, the theory of obsession seemed to be the most prevalent psychological explanation. The theory of resistance to social norms was the most frequently mentioned among the social explanations presented in Wattel’s review article. This theory appeared to be applicable in several adolescent and adult cases in Japan as well.

## 4. Discussion

### 4.1. Gender Identity Development in Autistic Adolescents

Erik Erikson, a developmental psychologist, proposed a theory of psychosocial development that consists of eight stages throughout the human lifespan [69]. In Erikson’s theory of psychosocial development, adolescence is a period for establishing identity and the fifth stage of his theory is known as identity vs. role confusion, which typically occurs around ages 12 to 18. Based on Erikson’s theory, adolescence is a pivotal time for individuals to explore who they are, including their gender, social roles, and values. Adolescents typically go through a process of self-exploration, during which they experiment with different identities, including their gender identity, in order to develop a cohesive sense of self. Gender identity is established through interactions with others during the adolescent period. Due to social impairments, it is often challenging for autistic adolescents to develop their identity during this period of life. In Japan, child and adolescent psychiatrists generally support ASD cases continuously from infancy through adolescence and into adulthood. If child and adolescent psychiatrists do not observe any symptoms suggestive of GD in the early developmental stage, they might be cautious or skeptical of later confirming a diagnosis of GD because the complaint about GD would be so sudden in the course of a long period of follow up [70].

Rapid-onset gender dysphoria (ROGD) is a phenomenon that can be observed among adolescents and young adults, particularly AFAB [70,71,72,73,74]. ROGD could be considered when adolescents suddenly claim to be GD without prior indications of discomfort with their assigned gender. The concept of ROGD is well known among many Japanese child and adolescent psychiatrists. They might be too cautious in diagnosing GD along with ASD because the treatment of GD may include irreversible surgical treatments such as mastectomy and sex reassignment surgery (SRS) [57]. Also, many health care professionals may hear complaints of GD symptoms that do not persist for long during their longitudinal support of ASD cases from childhood to adolescence.

In our survey of child and adolescent psychiatrists in Japan, we demonstrated that the number of experienced cases where one had been faced with gender-nonconforming symptoms per respondent was limited [18]. To compensate for the lack of actual case experience, it is necessary for this category of professionals to learn about ASD with GD and GD-related symptoms through published case reports.

Although there were many papers reporting the association between ASD and GD in English-language literature databases, the number of case reports was relatively small, and we included 13 case reports for review. This could have been due to the bias that the co-occurrence of ASD and GD was not considered worth reporting in an academic journal. Meanwhile, there were 11 case reports from Japan. The overall impression was that the authors of case reports were very cautious about diagnosing gender-nonconforming symptoms observed in ASD as GD following *DSM-5-TR* [75]. We speculate that the reason for the tendency to refrain from giving a diagnosis of GD for cases with GD-like symptoms in ASD could be that many of the gender-nonconforming symptoms might fall within the clinical features of ASD.

However, again, it should be noted that ASD is not a contraindication of gender conforming therapy, as the clinical guidelines clearly state [19]. The best treatment should be considered carefully according to the individuality of the case.

### 4.2. Fifteen Theories Proposed by Wattel

We tried to understand the underlying theories that connect ASD and GD. Regarding promising underlying theories of the association between ASD and GD, referring to the eight theories conceptualized by van der Miesen’s group [26], Wattel and her colleagues proposed fifteen theories, split between three categories [27]. Engel proposed a Bio-psychosocial (BPS) model to understand mental health issues with a wider range of approach [76]. In contrast to the traditional biomedical model, which mainly focuses on biological factors (such as genetics, neurobiology, and pathology), the BPS model incorporates biological, psychological, and social factors to provide a more comprehensive understanding of an individual’s condition. Wattel’s 15 theories are categorized into biological, psychological, and social factors [27]. Wattel’s 15 theories are summarized in Table 3.

When we reported a boy with ASD with comorbid GD in 2008 [50], theories explaining the comorbidity of these two conditions were limited to the extreme male brain theory [77]. At the time, ASD cases with a confirmed diagnosis of GD were limited to Landen’s case [45], Perera’s case [47], and Kraemer et al.’s case [49]. These were all AFAB ASD cases. Kraemer considered ASD with comorbid GD to be based on Baron-Cohen’s EMB and GD to be a result of the EMB. Since then, various studies have been published. After van der Miesen’s eight theories, Wattel et al. proposed fifteen theories that link ASD and GD. These theories are useful in understanding the co-occurrence of ASD and GD, especially in AMAB cases.

### 4.3. Gender Dysphoria and Gender-Nonconforming People in Japan

Here, we briefly review the findings about GD and gender identity-related concerns in Japan. With regard to the prevalence of GD, the *DSM-5* reported that Japan, along with Poland, was one of only two countries with a higher rate of AFAB compared to AMAB [1]. However, in *DSM-5-TR*, this description was deleted [75]. In ICD-11, the term gender identity disorder (GID) was replaced by gender incongruence (GI) [78]. GI was also moved from the category of disability (ICD-10: F00-99 Mental and behavioral disorder) to that of condition (ICD-11: 17 Conditions related to sexual health). Since GI is no longer considered a disability, it would be inappropriate for us to use the term “prevalence”.

Based on reports from medical institutes in Japan, with regard to the number of people who visited specialized gender clinics complaining of gender-nonconforming feelings, AMAB was consistently more common than AFAB in “GD in children” in *DSM-5*, while AFAB was dominant in “GD in adolescents and adults” [18,79,80].

### 4.4. Japanese School Culture and Psychological Burden Among Gender-Dysphoric Students

In general, school environments are based on the concept of a gender binary, which classifies one’s gender into two distinct and opposite categories: male and female. One of the most common problems faced by adolescents with GD in Japan is school uniforms. In the last decade or so, the introduction of genderless school uniforms has made it possible for students to select their uniforms (a skirt or pants) and wear a neutral design. Before then, in general, junior high and high school students were required to wear school uniforms based on their assigned gender, typically a skirt for girls and trousers for boys. They were also requested to obey school regulations related to hair length, though the strictness of these was highly school-dependent.

Going back even further, until the early 2000s, students would be made strongly aware of their gender upon entering elementary school. Up until then, it was the norm for girls to wear skirts and for boys to wear short pants at elementary school entrance ceremonies. In Japan, there is a custom to use a traditional, sturdy, and iconic school backpack, named *randoseru* in Japanese, during elementary school. In this context, there was a stereotyped, rigid idea that boys’ backpacks were black while girl ones were red, until colorful *randoseru* backpacks started becoming widely available in the early 2000s.

During childhood, feminine boys are more likely to be the target of bullying by classmates than boyish girls. In particular, children with typical development may hide their gender-dysphoric feelings in their daily lives.

### 4.5. The Present Rate of Gender-Nonconforming People in Japan

There are currently no reliable statistics on the occurrence of GD among adolescents in Japan. GD indicates a transgender person who has been diagnosed by a health professional for any reason. Therefore, we refer to the incidence of transgender people reported to date.

A survey of 2730 U.S. middle school students reported the occurrence of transgender students as 1.3% [81], while a survey of 8166 high school students in New Zealand reported a 1.2% occurrence [82]. A survey of LGBTQ+ persons among Japanese university students revealed the rate of transgenderism to be 0.8 [83]. As we noted earlier, the rate of transgenderism was 1.15% in the latest LGBTQ+ survey in Japan. Recently, the Gender Clinic of Okayama University Hospital performed a large-scale population-based online survey using the Utrecht Gender Dysphoria Scale (UGDS) [84,85]. Based on the results of this study, which combined self-acknowledged gender-dysphoric feelings and scores above the cutoff of 41 on the UGDS, it was demonstrated that the rate of possible GD was 0.87% for those who self-identified as male and 1.1% for those who self-identified as female. Taken together, it is plausible to estimate the rate of transgenderism to be around 1% among Japanese adolescents. In Japan, a private marketing company conducted a survey about lesbian, gay, bisexual, and transgender people, alongside those questioning and others (LGBTQ+), in a community sample. The latest survey conducted in 2023 estimated the total number of LGBTQ+ people in Japan to be 9.7% (group 2023). In more detail, the reported rates were 1.01%, 1.59%, 3.20%, and 1.15% for L, G, B, and T, respectively.

In 2013, a national survey of schools by the Ministry of Education, Culture, Sports, Science and Technology (MEXT) revealed that at least 606 students in primary, junior high, and high school considered themselves to be GD, and about 60% of them received some form of special treatment at school such as special accommodations for toilets, changing rooms, school uniforms, bed assignments during field trips, and swimming classes [86]. It should be noted that this number was limited to cases that the school was aware of and that the student had agreed to report. In reality, it is highly likely that considerably more students with GD exist in those populations.

### 4.6. “Diagnostic and Treatment Guidelines for Gender Incongruence” in Japan

In Japan, the treatment of GD strictly follows the “Diagnostic and Treatment Guidelines for Gender Incongruence” (in this paper, we refer to it as “Japanese GD guidelines”), as established by the Committee on Gender Dysphoria, the Japanese Society of Psychiatry and Neurology, and the Japanese Society of Gender Incongruence [87]. These guidelines were originally developed in accordance with SOC by WPATH [57]. The latest edition of the Japanese GD guidelines is found in the fifth edition and was published in August 2024.

The new Japanese GD guidelines emphasize that, when assessing and supporting GD in childhood, it should be noted that such dysphoria does not necessarily persist in adulthood. However, it has been reported that school refusal and self-injurious behavior may be observed among gender-dysphoric children. Therefore, while taking into consideration the possibility that GD in childhood may not persist, it is necessary to listen to any complaints from gender-dysphoric children and provide an environment in which they can verbalize their feelings of discomfort arising from gender nonconformity. Even if the children do not continue to meet the diagnostic criteria for GD, providing continuous support is necessary.

It has also been reported that gender-dysphoric children often have psychiatric comorbidities such as mood disorders, anxiety disorders, various kinds of trauma, eating disorders, autistic spectrum characteristics, suicidal ideation, and self-harm behaviors. As for psychological trauma, gender-dysphoric feelings may occur as a manifestation of inadequate parenting by guardians or experiences of being abused, especially sexual abuse.

The new guidelines underline that preoccupation and obsession with opposite-gender roles and behaviors among autistic people may be related to GD. However, regardless of whether GD is an expression of these comorbid conditions or has impact on these comorbid conditions, it is nonetheless important to carefully assess such feelings of discomfort and take measures to mitigate them so that the child does not feel emotionally overburdened. This is very important for the cultivation of identity in adolescents.

## 5. Conclusions

There is much interest in the comorbidity of ASD and GD. In 2018, the initial clinical guidelines for co-occurring ASD and GD in adolescents were published, suggesting the increasing need for intervention around these conditions [19]. Many studies on these themes have been published, including studies investigating GD and related symptoms in ASD and others investigating ASD comorbidity and ASD traits in GD. However, it is still unknown which factors, if any, link these two, often comorbid, conditions. Since the numerous reviews published to date have only included articles published in English, in this review paper, we investigated cases reported in Japanese. Further studies are awaited.

## Figures and Tables

**Table 1:** Cases reported in English.

Author(s)	Year	Country	Age	Assigned Gender	Diagnosis	Potential Applicable Theories
Williams et al. [44]	1996	US	3	AMAB	ASD with cross-gender preoccupations	Gender development Obsessions
			5	AMAB	ASD with cross-gender preoccupations	Gender development Obsessions
Landen & Rasmussen [45]	1997	Sweden	14	AFAB	ASD with feelings of GD	Extreme male brain Feeling different Social communication
Mukaddes [46]	2002	Turkey	7	AMAB	ASD with gender identity problems	Gender development Social communication
			10	AMAB	ASD with gender identity problems	Gender development Social communication
Perera [47]	2003	Sri Lanka	9	AFAB	ASD, GD and OCD	Extreme male brain Obsessions
Gallucci et al. [48]	2005	US	adult	AMAB	ASD, GD	Obsessions Social communication
Kraemer et al. [49]	2005	Switzerland	adult	AFAB	ASD, GD	Extreme male brain Resistance to social norms
Tateno et al. [50]	2008	Japan	5	AMAB *	ASD, GD	Gender development Social communication
Jacobs et al. [52]	2014	US	adult	AMAB	ASD, GD	Rigidity ToM/mentalizing Resistance to social norms
			adult	AMAB	ASD, GD	Rigidity ToM/mentalizing Resistance to social norms
Lemaire et al. [53]	2014	France	adult	AFAB	ASD, GD	Feeling different Resistance to social norms
Parkinson [54]	2014	Australia	adult	AMAB	ASD with gender identity problems	Rigidity Resistance to social norms
			adult	AMAB	ASD with gender identity problems	Rigidity Resistance to social norms
Tateno et al. [51]	2015	Japan	16	AMAB *	ASD (GD no longer meets diagnostic criteria)	Social communication
Zupanic et al. [55]	2021	Slovenia	16	AFAB	ASD, GD	Obsessions Feeling different Resistance to social norms
Sheikhmoonesi [56]	2023	Iran	adult	AMAB	ASD, GD	Feeling different Resistance to social norms

AFAB: assigned female at birth; AMAB: assigned male at birth; ASD: autism spectrum disorder; GD: gender dysphoria; and OCD: obsessive–compulsive disorder. * indicates the same case, while the term adult means someone who is 18 years of age or older.

**Table 2:** Cases reported in Japanese.

Author(s)	Year	Country	Age	Assigned Gender	Diagnosis
Kanbayashi [61]	1996	Japan	9	AMAB	ASD with gender identity problems
Kato and Ishida [62]	2002	Japan	adult	AMAB	ASD with crossdressing
Goto et al. [59]	2005	Japan	adult	AMAB	ASD with gender identity problems
Tateno and Tateno [67]	2008	Japan	7	AMAB *	ASD, GD
Tateno et al. [65]	2011	Japan	14	AFAB	ASD with gender identity problems
			15	AMAB #	ASD with crossdressing
			16	AMAB $	ASD with homosexual
			5	AMAB *	ASD, GD
Itoh and Yamato [58]	2013	Japan	adult	AMAB	ASD with feelings of GD
Tateno and Ikeda [64]	2017	Japan	13	AFAB	ASD with feelings of GD
			16	AMAB $	ASD with homosexual
			14	AMAB	ASD with crossdressing like behavior
			15	AMAB #	ASD with crossdressing
			16	AMAB	ASD with gender identity problems (Questioning)
Yamaguchi [68]	2018	Japan	adult	AMAB	ASD with feelings of GD
Mori and Tanebe [63]	2020	Japan	13	AMAB	ASD with X-gender

AFAB: assigned female at birth; AMAB: assigned male at birth; ASD: autism spectrum disorder; and GD: gender dysphoria. *, #, and $ indicate the same case, while adult means someone who is 18 years of age or older.

**Table 3:** Theories that link ASD and GD proposed by Wattel.

Theories	Brief Description
**Biological factor**	
Prenatal hormones	Intrauterine androgen exposures may contribute to ASD and GD
Extreme male brain theory	An exaggeration of typical male cognitive traits in AFAB (“Systemizing” is superior to “empathizing” in ASD)
Birthweight	High birthweight could be related to both ASD and GD
Genetic factors	Common genetic predisposition for ASD and GD
**Psychological factor**	
Obsessions	Obsessions may make ASD appear as GD
Rigidity	Cognitive inflexibility in ASD causes more rigid and concrete thinking about gender that may result in occurrence of GD
Gender development	Atypical gender development in ASD may lead to GD
Theory of Mind (ToM)/mentalizing	Deficits in ToM/mentalizing abilities in ASD may lead to GD
Weakened sex differences	GD in ASD may be due to weakened sex differences
Sexual orientation	ASD may assume to be of another gender when sexually attracted to the same gender
Sensory processing	Over- or under-responsivity to specific sensory stimuli is a commonfactor in ASD and GD
**Social factor**	
Resistance to social norms	GD is more common among ASD compared to neurotypical people because they are less susceptible to social norms/prejudice
Minority stress	ASD traits in GD may reflect social difficulties due to GD
Feeling different	GD in ASD is associated with feeling different from others in their assigned gender group
Social communication	Deficits in social communication due to ASD result in little understanding of gender norms

ASD: autism spectrum disorder; and GD: gender dysphoria. The bold text indicates the three factors of Bio-Psycho-Social model.

**Table 4:** Brief descriptions of Japanese cases and applicable theories.

Author(s)	Year	Diagnosis	Brief Description of Cases	Theories
Kanbayashi [61]	1996	ASD with gender identity problems	An AMAB ASD case followed for approximately 20 years. During puberty, he exhibited homosexual-like behavior. He was confused by his secondary sexual characteristics, but was convinced that his biological sex was male. Because he was bullied by other boys, he thought that girls were peaceful and felt identical to girls. Kanbayashi considered his gender identity confusion as one aspect of ASD features.	Gender development Sexual orientation Theory of Mind Feeling different
Kato and Ishida [62]	2002	ASD with crossdressing	An adult AMAB ASD with crossdressing. He preferred wearing skirts to feel satisfied and calmed him down. On the other hand, he did not report any gender non-conforming feelings or desire to be female. Crossdressing was considered an obsession with a specific feeling or a limited object.	Obsessions (restricted interests)
Goto et al. [59]	2005	ASD with gender identity problems	An adult AMAB case said he wanted to be a female. But no cross-dressing, no desire to be reassigned to female, and no strong and persistent identification with the opposite gender, no persistent discomfort and inappropriateness with his assigned gender were observed. Goto et al. stated that the all symptoms related to GD could be explained by autistic traits. As the females were kind and accepting in contrast to the males who repeatedly bullied, he identified himself as female.	Gender development Feeling different Social communication
Tateno and Tateno [67]	2008	ASD, GD	An AMAB ASD in childhood with comorbid GD. The difficulties in social communication and gender development seemed to be affecting the occurrence of GD. Please refer to the case reports in Englich. GD symptoms were alleviated.	Gender development Social communication
Tateno et al. [65]	2011	ASD with gender identity problems	An AFAB ASD in adolescent who insisted on GD and preoccupied to changing body by mastectomy. She had no desire to become a male and insisted only on receiving mastectomy. The idea that breasts should not be found in anyone except female was uncorrectable. She did not attend junior high school and refused to ware school uniform for female.	Obsessions (restricted interests) Rigidity Social communication
Tateno et al. [65]	2011	ASD with crossdressing	Fifteen-year-old AMAB ASD loved the anime entitled PreCure© that features young girls who transform into magical heroes to fight evil. He liked to wear a PreCure© apron for young girls and was once warned by a school teacher for wearing it at junior high school. His grandmother was concerned about her grandson’s behaviors because he often played with his 4-year-old cousin girl pretending PreCure©. His grandmother brought him to the child and adolescent psychiatry clinic to exclude GD.	Obsessions (restricted interests) Theory of Mind Social communication
Tateno et al. [65]	2011	ASD with homosexual	An adolescent AMAB ASD was severely bullied by a group of females at school repeatedly. He was afraid of females and stated that he did not want to even get close to them. He confessed to the authors that he had romantic feelings for a classmate of the same assigned gender who helped him always. He was concerned that he was different from typical male and he could be GD because he loves the boy.	Feeling different Sexual orientation
Itoh and Yamato [58]	2013	ASD with feelings of GD	An adult AMAB ASD who insisted on GID of DSM-IV-TR but did not meet the diagnostic criteria. The crossdressing was considered to be due to an excessive preoccupation with Gothic and Lolita fashion, so-called GothLoli, which is a distinctive subculture of Japanese street fashion. Deficits in social communication might have made it difficult for him to adjust to assigned gender group.	Obsessions (restricted interests) Social communication
Tateno and Ikeda [64]	2017	ASD with feelings of GD	An adolescent AFAB ASD had difficulty adjusting to same gender groups resulting in school refusal. She was isolated and stated that classmates of same assigned gender were very different from her as if they were from a different world. She said she might have GD.	Feeling different Social communication
Tateno and Ikeda [64]	2017	ASD with crossdressing like behavior	An AMAB ASD in adolescent liked to wear women’s pantyhose, saying that the tightness and smoothness to the touch were the best. One day at junior high school, when he was changing clothes, his friends caught sight of him and made fun of him. But he did not care. He said he also liked the fluffy feeling of skirts and confessed that he also wore skirts at home.	Obsessions (restricted interests) Sensory processing
Tateno and Ikeda [64]	2017	ASD with gender identity problems (Questioning)	The case was a thin AMAB ASD with feminine features. His skin was quite smooth. He shaved his arms and legs and took female hormones that he had obtained from a private importer. He insisted that he did not want to become an adult male because he was not biological male and hated secondary sexual characteristics and masculinization. He emphasized that he was gender-neutral and that he did not agree with the concept of binary.	Weakened sex different Resistance to social norms
Yamaguchi [68]	2018	ASD with feelings of GD	An AMAB ASD who was confused by secondary sexual characteristic. He presented serious confusion in the development of gender identity. The case complained that the skin sensation on his extremities by body hair was terribly uncomfortable.	Gender development Sensory processing
Mori and Tanebe [63]	2020	ASD with X-gender	An adolescent AMAB ASD complaining X-gender. The case’s suicidal ideation was alleviated by suppression of secondary sexual characteristics with female hormones (patch). The authors explained that complaint of X-gender in ASD may be related to an ambiguous self-image.	Gender development Resistance to social norms

AFAB: assigned female at birth; AMAB: assigned male at birth; ASD: autism spectrum disorder; and GD: gender dysphoria. Adult means someone who is 18 years of age or older.
